# Nitric oxide is associated with fracture risk in Japanese women

**DOI:** 10.1371/journal.pone.0280854

**Published:** 2023-02-07

**Authors:** Masataka Shiraki, Tatsuhiko Kuroda, Masaki Nakano, Yukio Nakamura, Mitsuru Saito, Tomohiko Urano

**Affiliations:** 1 Research Institute and Practice for Involutional Diseases, Azumino City, Nagano, Japan; 2 Public Health Research Foundation, Shinjuku-ku, Tokyo, Japan; 3 Department of Orthopaedic Surgery, Shinshu University School of Medicine, Matsumoto City, Nagano, Japan; 4 Department of Orthopaedic Surgery, Jikei University School of Medicine, Minato-ku, Tokyo, Japan; 5 Department of Geriatric Medicine, International University of Health and Welfare School of Medicine, Narita City, Chiba, Japan; Medical College of Wisconsin, UNITED STATES

## Abstract

Although nitric oxide (NO) is a known factor that regulates the bone physiology, few and discordant results have been obtained in human studies evaluating the effect of nitrates on bone health. We investigated for the relationship between serum NOx level and incident osteoporotic fracture rate prospectively in a cohort consisting of Japanese women. A total of 871 subjects (67.5 ± 10.8 y/o) were analyzed. During the observation period (8.8 ± 7.2 yrs), incident osteoporotic fractures occurred in 267 participants (209 vertebral fractures, 57 long-bone fractures, and 1 both types). Hazard ratio, by the Cox proportional hazards model, of serum NOx for incident fracture was 0.64 (95% confidence interval 0.53–0.78, p < 0.001) after adjustment for baseline age (1.13, 1.06–1.21, p < 0.001), lumbar bone mineral density (L-BMD; 0.85, 0.78–0.92, p < 0.001), presence of prevalent fracture (3.27, 2.49–4.32, p < 0.001), and treatment of osteoporosis (0.70, 0.53–0.92, p = 0.010). The relationships between serum level of NOx and bone-related parameters were examined by multiple regression analysis; body mass index (p < 0.001) and L-BMD (p = 0.011) were significantly associated with serum NOx level. These results suggest that the low circulating NOx is one of the independent predictors for osteoporotic fracture occurrence in postmenopausal women.

## Introduction

Osteoporosis is characterized by the deterioration of bone strength, which leads to high fracture susceptibility. Osteoporotic fractures cause a decline in activities of daily living (ADLs), a long-term treatment or care, and the ensuing socio-economic burden. Since osteoporosis principally affects postmenopausal women, estrogen deficiency would play a central role in its pathogenesis through a facilitation of osteoclast differentiation and activity. The signal of estrogen deficiency is mediated by the receptor activator of NF-κB ligand (RANKL), which is expressed in osteoblasts and osteocytes. RANKL binds to a physiological receptor, RANK, on the osteoclast lineage cells and induces osteoclast differentiation. It has been accepted that the activation of RANKL-RANK system after menopause is responsible for the excess bone resorption [1]. Inhibition of RANKL-RANK system by the specific antibody for RANKL, denosumab, has been widely utilized in the treatment of osteoporosis with successful fracture prevention [2]. On the other hand, bone forming activity brought by osteoblasts is relatively decreased after menopause. Osteoblast differentiation and activity are regulated by Wnt signaling and several growth factors [3]. The balance of bone resorption and formation tends to be negative after menopause, thereby leading to a bone loss. In addition to RANKL-RANK system and Wnt signaling, nutritional, genetic, or lifestyle-dependent background influence the bone metabolic balance.

A variety series of chemical substances or local factors may modulate the bone remodeling system. Collin-Osdoby et al. proposed a significant role of nitric oxide (NO) in the skeletal physiology, especially in the bone remodeling, about 30 years ago [4]. NO is produced in trace quantities in neurons, endothelial cells, neutrophils, and bone cells through the oxidation of arginine by nitric oxide synthase (NOS). As a recipient of oxygen radicals, the NO generated in various tissues is immediately converted into the final products, nitrite (NO_2_^−^) or nitrate (NO_3_^−^) [5]. NOS has been identified in osteoblasts and osteocytes. The mechanical stress signals are transduced to NOS expression in osteocytes [6, 7]. In vitro studies demonstrated that NO inhibited osteoclastic activities [4]. In order to elucidate the action of NO on bone metabolism in vivo, NOS knockout (KO) mice have been generated. Three types of NOS isozyme have been identified so far, namely, neuronal NOS (nNOS or NOS-1), inducible NOS (iNOS or NOS-2), and endothelial NOS (eNOS or NOS-3). The phenotype of NOS-1 KO mouse exhibited osteosclerosis owing to the decrease in number of osteoclasts. NOS-2 KO mouse presented no obvious phenotype in bone, but showed a decrease in reloading induced osteogenesis [8]. Reduced bone length, bone volume, and bone mineral density (BMD) were observed in NOS-3 KO mouse. NOS-3 KO mouse showed no excess bone resorption after ovariectomy [9]. On the other hand, Cuzzocrea et al. reported that estrogen depletion resulted in an over-production of NO with enhanced serum levels of inflammatory cytokines. Diminished iNOS activity in iNOS KO mice (iNOS^(−/−)^) led to no bone loss after ovariectomy, indicating that the estrogen-deficiency signal might be mediated by NO production [10]. Although the targeting enzymes were different in these reports, the modes of NO action on bone metabolism seemed to be discordant.

Three intervention studies on BMD using nitroglycerin as a source of NO have been reported. The first report investigated the effects of intermittent use of nitrates on BMD and fracture incidence in postmenopausal women [11]. The intermittent nitrate users exhibited a greater BMD compared to non-users; however, there had been no risk reduction in osteoporotic fractures, possibly due to the lack of statistical power [11]. The second report appeared in 2000 and showed that nitroglycerin treatment was effective to the same extent as estrogen administration for reducing bone loss after oophorectomy [12]. Very recently, Bolland et al. carried out a randomized controlled trial targeting the effects of nitrates on BMD and bone turnover markers (BTMs) in postmenopausal women with osteopenia [13]. This trial resulted in a negative study since nitrate use demonstrated no beneficial effects on both BMD and BTMs. Judging from these clinical trial results, the effectiveness of nitroglycerin use for the treatment of osteoporosis seems to be discordant.

Until now, however, there have been no available data regarding the relationship between circulating NOx levels and bone status in humans. To confirm the possible rationale for supplementation of nitrates to prevent fractures, we planned to explore whether the associations of NOx with bone status or fracture susceptibility exist or not. If the plausible association between NOx level and bone status would be certified by the clinical studies, it may open the new corridor to understand bone response against mechanical stress or pathophysiology of osteoporotic fracture. In the present study, we investigated for the associations of serum NOx level with BMD and incident osteoporotic fracture rate in a cohort consisting of Japanese women.

## Materials and methods

### Subjects

Subjects of this study were a subset of enrolled participants into the Nagano cohort study, which is an ongoing registration study of peri- or post-menopausal female outpatients visiting at a primary care institution in Nagano Prefecture, Japan, since 1993.

As shown in our previous studies [14–18], the consecutive patients who were measured BMD and assessed prevalent bone fractures were registered in the Nagano cohort study after obtaining written informed consent. After registration, the subjects conflicting with exclusion criteria, such as patients with secondary osteoporosis, critical illness, terminal stage of malignancy or chronic obstructive lung disease, long-term steroid use, or nitroglycerine users, were excluded from the present investigation. Terminal stage of chronic renal failure subjects whose estimated glomerular filtration rate (eGFR) was less than 20 mL/min/1.73m^2^ were also excluded. The previous our studies presented that no selection bias was noticed in terms of patient body size or BMD compared to the community-dwelling Japanese population by using the propensity score. Follow-up was terminated by the occurrence of first incident osteoporotic fracture, death of the subject, or discontinuation of follow-up due to moving, institutionalization, or reference to another hospital, whichever happened.

The protocol of this study was reviewed by the ethics committee of the Research Institute and Practice for Involutional Diseases, Japan, prior to commencement and was conducted in accordance with the principles of the Declaration of Helsinki. Comprehensive written informed consent was obtained from all subjects.

### Bone parameters and NOx measurement

Serum level of NOx (NO_2_^−^ and NO_3_^−^) was measured by colorimetric assay kit (Cayman Chemical, MI, USA) using Griess reagent. The inter- and intra-assay variances in the laboratory were 2.7% and 2.1%, respectively. In addition, we had checked intra-assay variance of serum samples obtained from 2 different time points in 10 participants. The coefficient of variation (CV) was 14.2 ± 9.2% (mean ± standard deviation). BMD at L2 to L4 lumbar spine (L-BMD) was determined by dual energy X-ray absorptiometry (DPX series, GE Healthcare, IL, USA). Serum levels of parathyroid hormone (PTH) and 25-hydroxyvitamin D (25[OH]D) were measured by electrochemiluminescence immunoassay (ECLIA). As for BTMs, serum level of bone-derived alkaline phosphatase (BAP; Ostase CLIA, Beckman Coulter, CA, USA) and urinary excretion of type I collagen cross-linked N-telopeptides (NTx; Osteomark, Creative Diagnostics, NY, USA) were measured. Serum homocysteine level was determined by using high-performance liquid chromatography (HPLC). Urinary excretion of pentosidine was measured by HPLC system after hydrolysis of urine samples [16, 17]. All the biochemical parameters listed above were assayed at the central laboratory (LSI Medience, Tokyo, Japan).

### Fracture evaluation

Prevalent fractures were defined as non-traumatic fractures occurring in vertebrae, ribs, pelvis, proximal end of the humerus, distal end of the radius, clavicula, proximal end of the femur, or proximal or distal end of the tibia or fibula. Participants were asked about their history of long-bone fractures in interviews, or their medical records were checked. Although some cases of prevalent long-bone fracture could not distinguish traumatic and fragility fracture, because of the loss of memory or medical record, the history of long-bone fracture was accounted a prevalent fracture. Vertebral fractures were diagnosed by semi-quantitative analysis using X-ray films of the thoracic and lumbar spine at the baseline [19]. Incident vertebral fractures were evaluated by X-ray films taken every 1 to 2 years interval, with additional X-ray assessment on demand. Incident long-bone fractures were confirmed by the clinical symptoms with X-ray film evaluation, the medical record, or the memory of participants. The incident long-bone fractures after receiving major trauma were excluded, while fragility fractures caused by minor trauma were defined as osteoporotic fractures.

### Biochemical data

Serum and urine samples were collected at baseline to determine biochemical markers. Serum levels of albumin, total cholesterol, triglycerides, creatinine, calcium, and phosphate were assayed by the in-house laboratory using dry chemical system (Dry Chem Series, Fujifilm, Tokyo, Japan). Glycated hemoglobin (HbA1c) was measured by HPLC (HLC-723 G series, Tosoh, Tokyo, Japan). The eGFR was calculated by the formula indicated below:

eGFR = Age^−0.287^ × Cr^−1.094^ × 194 × 0.739.

Serum levels of adiponectin and high-sensitive C-reactive protein (hsCRP) were measured by Latex kit (LSI Medience) and N-Latex CRP II (CardioPhase hsCRP, Siemens Healthineers, Bayern, Germany), respectively. Serum leptin level was determined by leptin radioimmunoassay (RIA) kit (Merck Millipore, MA, USA). These biochemical parameters were assayed at LSI Medience.

### Definition of co-morbidities

The prevalence of co-morbidities, including diabetes mellitus (DM), dyslipidemia, hypertension, vascular events, and past history of cancer (cancer survivor), was determined by the diagnostic criteria of each disease and/or relevant treatment records as we described previously [14]. The diagnostic criteria for these co-morbidities are described as follows: DM was diagnosed if hemoglobin A1c level was ≥ 6.5% or when the patient was actively treated for DM. All the patients with diabetes were type II diabetes and the patients with insulin treatment were also included. Dyslipidemia was defined as low-density lipoprotein-cholesterol ≥ 140 mg/dL, high-density lipoprotein-cholesterol < 40 mg/dL, or postprandial triglycerides ≥ 200 mg/dL. Hypertension was diagnosed when systolic blood pressure was persistently > 140 mmHg, diastolic pressure was persistently > 90 mmHg, or anti-hypertensive drugs were used. The prevalence of vascular events was defined as having clinically diagnosed cerebrovascular or ischemic heart disease, which were diagnosed by brain computed tomography (CT) or magnetic resonance imaging (MRI) with clinical sign for the cerebrovascular disease and by multi-detector CT or coronary angiography for the ischemic heart disease. Multiple lacunar findings in the brain were not counted as the cerebrovascular disease. Survivors from any kinds of malignant tumor in their medical history at baseline were defined as the participants with cancer.

### Treatment

All morbid states during observation as well as at baseline were properly treated over the follow-up period after obtaining informed consent from the participants. All subjects with osteoporosis were asked for receiving osteoporotic treatment. When the patients agreed with treatment, the patients themselves selected the modes of treatment with the advice from clinicians. Definition of the treatment was as follows. The mode of treatment included teriparatide, estrogen, selective estrogen receptor modulators (SERMs), bisphosphonates, or denosumab. When the treatment term exceeded half of the observation period, we defined those subjects as the patients with treatment. Vitamin D analogue, vitamin K2, calcium formulation, or calcitonin were not included in the treatment because of the lack of robust data for preventing fractures. Randomization of the treatment mode was impossible, because the treatment procedure depended upon the patient’s will. The treatment of osteoporosis was basically not changed until the occurrence of incident fracture or adverse event. Thus, the duration of treatment was the same as observation period in nearly all cases.

### Statistical analysis

Numerical data are presented as the mean ± standard deviation (SD). Categorical data are indicated by the number and proportion of subjects. Since the distribution of serum NOx did not adapt to Gaussian distribution, log-transformed NOx values were applied to the following analysis. The baseline bone parameters including serum NOx level were compared between the subjects with and without incident osteoporotic fractures by using *t*-test or χ^2^ test. In the longitudinal analysis, the Cox proportional hazards model was employed to calculate the hazard ratios (HRs), with 95% confidence interval (CI), for the fracture occurrence with adjustment for traditional risk factors and potential confounding factors, which were selected based on the previous studies [14–18]. In the present investigation, the adjustment factors were selected from the parameters which were significantly different between the groups with and without incident fractures. Kaplan–Meier curves were plotted to delineate the incidence of fractures over the observation period, and the log-rank testing was used to examine the significance of differences among the groups divided by the quartiles of circulating NOx. The relationships between serum NOx level and bone parameters were evaluated by ANOVA and multiple regression analysis. All comparisons were two-sided, and p value of < 0.05 was considered statistically significant. The data were analyzed using JMP version 16.0 software (SAS Institute, NC, USA).

## Results

Serum levels of NOx were determined in a total of 871 participants by the colorimetric method. The mean ± SD duration of follow-up was 8.8 ± 7.2 years. Baseline data including bone and metabolic parameters and co-morbidities are presented in Table 1.

**Table 1:** Baseline data of total subjects and subjects with or without incident osteoporotic fractures.

Item (n)	Total (871)	Incident fracture		P
		Yes (267)	No (604)	
Age, years	67.5 ± 10.8	72.3 ± 9.5	65.4 ± 10.7	< 0.001
BMI, kg/m^2^	22.4 ± 3.4	22.5 ± 3.4	22.4 ± 3.3	0.760
L-BMD, g/cm^2^	0.936 ± 0.196	0.871 ± 0.182	0.964 ± 0.196	< 0.001
Albumin, g/dL	4.2 ± 0.4	4.1 ± 0.3	4.3 ± 0.4	< 0.001
eGFR, mL/min/1.73m^2^	68.7 ± 18.5	66.7 ± 17.9	70.0 ± 18.8	0.030
Calcium, mg/dL	9.3 ± 0.5	9.2 ± 0.5	9.4 ± 0.5	< 0.001
Phosphate, mg/dL	3.5 ± 0.5	3.5 ± 0.5	3.5 ± 0.5	0.456
PTH, pg/mL	41.1 ± 18.1	38.3 ± 18.9	42.3 ± 17.6	0.005
25(OH)D, ng/mL	20.1 ± 5.9	20.4 ± 5.6	20.0 ± 6.0	0.470
Total cholesterol, mg/dL	207.6 ± 35.6	201.5 ± 35.9	210.3 ± 35.2	< 0.001
Triglycerides, mg/dL	145.0 ± 86.1	143.9 ± 75.8	145.5 ± 90.3	0.801
HbA1c, %	5.6 ± 0.8	5.5 ± 0.8	5.6 ± 0.8	0.105
NTx, nmoleBCE/mmoleCr	50.8 ± 30.6	53.2 ± 29.0	49.8 ± 31.3	0.144
BAP, U/L	31.1 ± 13.9	32.1 ± 13.4	30.5 ± 14.2	0.225
hsCRP, mg/dL	0.10 ± 0.21	0.10 ± 0.16	0.11 ± 0.23	0.761
Pentosidine, pmole/mgCr	46.3 ± 30.9	52.5 ± 39.2	43.2 ± 25.3	< 0.001
Homocysteine, nmole/mL	9.3 ± 3.6	9.8 ± 4.3	9.0 ± 3.2	0.009
Log-NOx, log, μmole/L	4.5 ± 0.6	4.4 ± 0.6	4.6 ± 0.6	< 0.001
Prevalent fracture, yes (%)	279 (32.0)	163 (61.0)	116 (19.2)	< 0.001
Diabetes mellitus, yes (%)	129 (14.8)	39 (14.6)	90 (14.9)	0.910
Hypertension, yes (%)	406 (46.6)	148 (55.4)	258 (42.7)	< 0.001
Dyslipidemia, yes (%)	412 (47.3)	118 (44.2)	294 (48.7)	0.222
Vascular events, yes (%)	61 (7.0)	26 (9.7)	35 (5.8)	0.041
Cancer survivor, yes (%)	58 (6.7)	23 (8.6)	35 (5.8)	0.132

Data are expressed as the mean ± SD or number (percent). The significance of differences between the values in patients with and without fractures were calculated by t-test or χ^2^ test. Post prandial serum or urine samples were obtained.

BMI, body mass index; L-BMD, lumbar bone mineral density; eGFR, estimated glomerular filtration rate; PTH, parathyroid hormone; 25(OH)D, 25-hydroxyvitamin D; HbA1c, glycated hemoglobin A1c; NTx, type I collagen cross-linked N-telopeptides; BAP, bone-derived alkaline phosphatase; hsCRP, high-sensitive C-reactive protein; NOx, nitrogen oxide.

The definitions of co-morbidities are presented in the Methods section.

Prevalent osteoporotic fractures were observed in 279 subjects (32.0%). A total of 386 patients (44.3%) had received the treatment of osteoporosis mainly by bone resorption inhibitors. Drugs and cumulative number of patients were conjugated estrogen (n = 18), SERMs (n = 81), bisphosphonates (n = 228), denosumab (n = 91), and teriparatide (n = 12). Bisphosphonate treatment included etidronate, alendronate, risedronate, minodronate, and zoledronate. During the observation period, 267 (209 vertebral fractures, 57 long-bone fractures, and 1 case experienced both types of fracture simultaneously) osteoporotic fractures occurred. We divided the subjects into two groups, with or without incident osteoporotic fracture, and the baseline data of those were compared (Table 1). The subjects with incident fracture exhibited significantly higher age (p < 0.001), urinary pentosidine (p < 0.001), and homocysteine (p = 0.009). The proportion of patients with prevalent osteoporotic fracture in the incident fracture group was 61.0%, while that in the non-incident fracture group was 19.2% (p < 0.001). On the other hand, fractured subjects displayed significantly lower NOx (p < 0.001), L-BMD (p < 0.001), albumin (p < 0.001), eGFR (p = 0.030), calcium (p < 0.001), PTH (p = 0.005), and total cholesterol (p < 0.001) than those of the subjects without incident fracture occurrence. The patients with hypertension and history of vascular events exhibited significantly higher rates of incident fractures. Statistical difference was not found in fracture incidence between the presence and absence of osteoporosis treatment.

HR of NOx for incident osteoporotic fracture was 0.64 (95% CI 0.53–0.78, p < 0.001) after adjustment for traditional fracture risk factors such as baseline age (HR 1.13, 1.06–1.21, p < 0.001), L-BMD (HR 0.85, 0.78–0.92, p < 0.001), presence of prevalent fracture (HR 3.27, 2.49–4.32, p < 0.001), and treatment of osteoporosis (HR 0.70, 0.53–0.92, p = 0.010) (Table 2: Model 1).

**Table 2:** Cox proportional hazards model for the association between serum NOx and incident osteoporotic fracture.

Item	HR	95% CI		P	Model
Age in every 5 years up	1.13	1.06	1.21	< 0.001	Model 1
L-BMD, 0.1 g/cm^2^ up	0.85	0.78	0.92	< 0.001	
Prevalent fracture, yes	3.27	2.49	4.32	< 0.001	
Treatment of osteoporosis, yes	0.70	0.53	0.92	0.010	
Log-NOx, 1 up	0.64	0.53	0.78	< 0.001	
Calcium, 0.1 mg/dL up	1.48	1.28	1.72	< 0.001	Model 2
Log-NOx, 1 up	0.81	0.73	0.91	< 0.001	
Pentosidine, 1 pmole/mgCr up	1.02	1.00	1.03	0.036	Model 3
Log-NOx, 1 up	0.66	0.54	0.82	< 0.001	
Homocysteine, 1 nmole/mL up	1.04	1.00	1.09	0.046	Model 4
Log-NOx, 1 up	0.69	0.55	0.86	< 0.001	
Hypertension, yes	1.57	1.23	2.00	< 0.001	Model 5
Log-NOx, 1 up	0.56	0.46	0.67	< 0.001	
Vascular events, yes	1.59	1.03	2.33	0.026	Model 6
Log-NOx, 1 up	0.55	0.46	0.67	< 0.001	

The hazard ratio (HR), with 95% confidence interval (CI), of NOx for incident osteoporotic fracture was calculated by the Cox proportional hazards model with adjustment for potential confounders.

Model 1 was adjusted for traditional fracture risks, age, L-BMD, prevalent fracture, and treatment of osteoporosis.

Models 2, 3, 4, 5, and 6 were adjusted for serum calcium, urinary pentosidine, serum homocysteine, prevalence of hypertension, and history of vascular events in addition to Model 1, respectively.

Serum level of total cholesterol and eGFR value were not incorporated into the models, because HRs of these parameters were not statistically significant.

L-BMD, lumbar bone mineral density; NOx, nitrogen oxide

In the Model 2, NOx level was a statistically significant independent predictor of fracture after further adjustment for calcium (HR 1.48, 1.28–1.72, p < 0.001). After adjustment for confounders including pentosidine (HR 1.02, 1.00–1.03, p = 0.036: Model 3) or homocysteine (HR 1.04, 1.00–1.09, p = 0.046: Model 4), NOx was still significant. The same consequences were obtained after further adjusting for prevalence of hypertension (HR 1.57, 1.23–2.00, p < 0.001: Model 5) or history of vascular events (HR 1.59, 1.03–2.33, p = 0.026: Model 6) (Table 2). Osteoporosis treatment using bone resorption inhibitors was shown to be an independent confounding factor for reducing incident fracture risk. The other possible confounders such as eGFR, PTH, or total cholesterol were not significant predictors for incident fractures.

**Fig 1:**
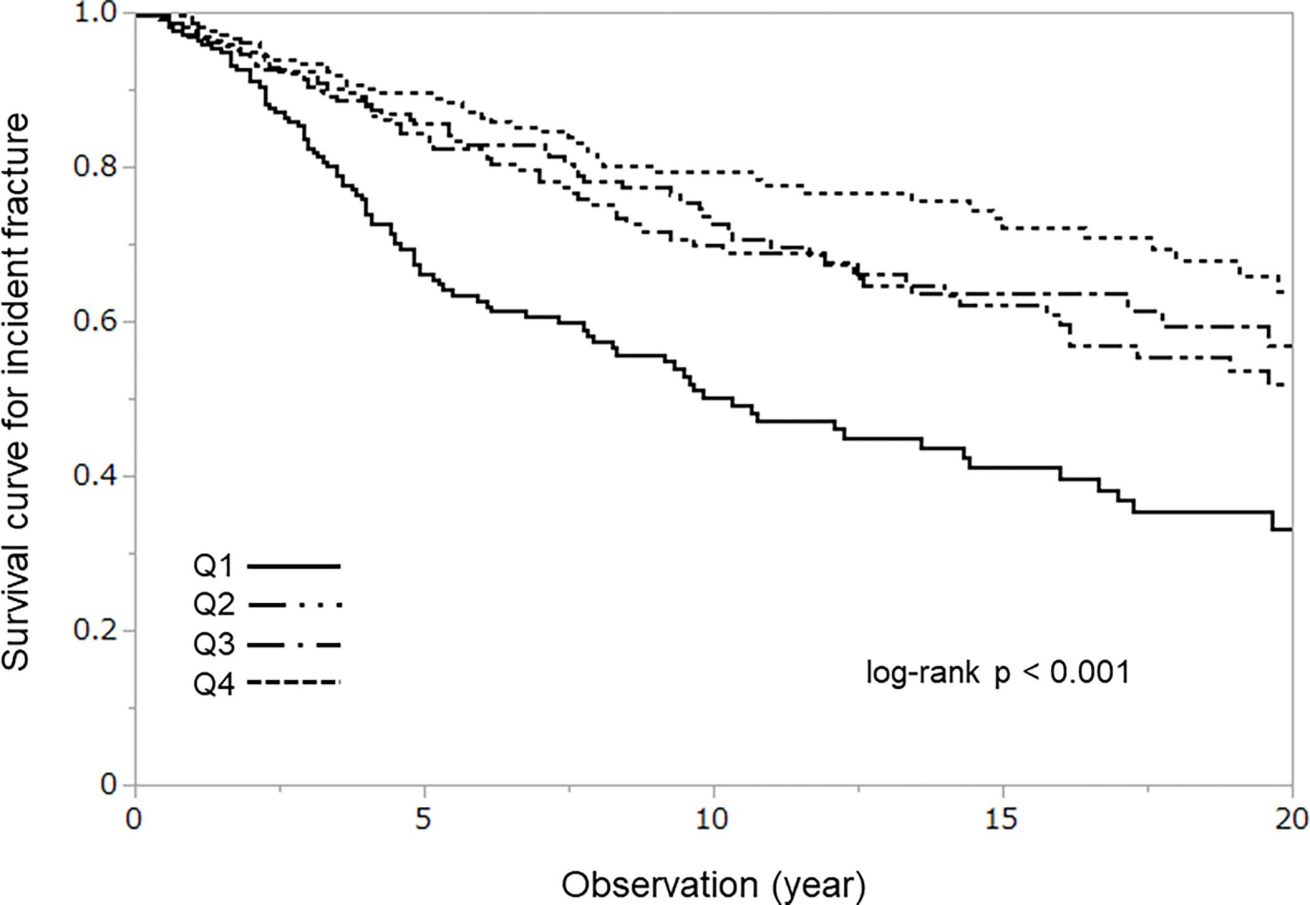
Time dependent fracture-free rates according to quartiles of the baseline NOx level. Subjects were divided into 4 groups according to the baseline serum level of NOx. The lowest group exhibited significantly higher and more rapid susceptibility to incident osteoporotic fracture than those in the other groups (p < 0.001 by log-rank test).

To examine the time-dependent fracture occurrence rate in the groups divided by the quartiles of serum NOx level, Kaplan–Meier plots were depicted in Fig 1. The group with the lowest NOx level demonstrated a significantly faster and higher frequency of incident fracture occurrence compared to the other quartiles (p < 0.001 by log-rank testing). The threshold of serum NOx for incident fracture occurrence obtained by receiver operating characteristic (ROC) analysis was 74.2 μmole/L.

To investigate the relationships among circulating NOx level, bone parameters, and co-morbidity prevalence, NOx quartile analysis for each item was performed. Table 3 indicated that serum level of NOx was significantly associated with body mass index (BMI), L-BMD, PTH, and urinary NTx levels. However, the baseline co-morbidity prevalence showed no significant correlations with serum NOx level.

**Table 3:** Quartile analysis of NOx for bone parameters and co-morbidities.

	NOx quartile (range [μmole/L], n)				
Item	Q1 (< 70.5, 257)	Q2 (70.5–104, 203)	Q3 (105–141, 210)	Q4 (141 <, 201)	P
Age, years	68.2 ± 11.1	68.6 ± 11.5	66.9 ± 10.5	66.3 ± 10.1	0.087
BMI, kg/m^2^	21.6 ± 3.2	22.1 ± 3.1	22.7 ± 3.5	23.2 ± 3.4	< 0.001
L-BMD, g/cm^2^	0.897 ± 0.190	0.926 ± 0.175	0.936 ± 0.204	0.983 ± 0.206	< 0.001
eGFR, mL/min/1.73m^2^	65.9 ± 17.5	70.7 ± 23.0	68.8 ± 16.2	70.0 ± 16.6	0.091
PTH, pg/mL	38.0 ± 19.1	42.5 ± 16.9	44.9 ± 21.3	38.9 ± 13.9	< 0.001
25(OH)D, ng/mL	20.5 ± 6.0	20.1 ± 6.1	19.2 ± 5.4	20.7 ± 6.0	0.099
NTx, nmoleBCE/mmoleCr	55.4 ± 31.2	49.1 ± 25.6	51.6 ± 29.6	47.4 ± 34.9	0.049
BAP, U/L	31.9 ± 12.4	28.9 ± 10.3	32.0 ± 13.6	31.7 ± 17.8	0.241
Pentosidine, pmole/mgCr	48.3 ± 26.2	48.3 ± 28.4	45.9 ± 22.5	42.9 ± 16.7	0.289
Homocysteine, nmole/mL	9.3 ± 3.7	9.3 ± 3.8	9.2 ± 3.7	9.2 ± 3.4	0.948
Hypertension, yes (%)	116 (45.1)	84 (41.4)	104 (49.5)	102 (50.7)	0.207
Vascular events, yes (%)	23 (8.9)	12 (5.9)	15 (7.1)	11 (5.5)	0.465

The associations of bone parameters and co-morbidities with quartiles of serum NOx (μmole/L) were evaluated by ANOVA and χ^2^ test, respectively.

BMI, body mass index; L-BMD, lumbar bone mineral density; eGFR, estimated glomerular filtration rate; PTH, parathyroid hormone; 25(OH)D, 25-hydroxyvitamin D; NTx, type I collagen cross-linked N-telopeptides; BAP, bone-derived alkaline phosphatase; NOx, nitrogen oxide

In order to determine the independence of these associations, multiple regression analysis was conducted. Among those parameters, BMI (p < 0.001) and L-BMD (p = 0.011) were shown to be the significant factors associated with serum NOx level (Table 4).

**Table 4:** Multiple regression analysis for serum NOx.

Item	Estimate	SE	P
BMI, kg/m^2^	0.0238	0.0071	< 0.001
L-BMD, g/cm^2^	0.3111	0.1224	0.011
PTH, pg/mL	0.0004	0.0013	0.738
NTx, nmoleBCE/mmoleCr	−0.0006	0.0008	0.442

SE, standard error; BMI, body mass index; L-BMD, lumbar bone mineral density; PTH, parathyroid hormone; NTX, type I collagen cross-linked N-telopeptides; NOx, nitrogen oxide

## Discussion

In the present study, we demonstrated that the serum NOx level was significantly associated with incident osteoporotic fractures after adjustment for possible confounders. Since serum level of NOx was significantly correlated with BMI and L-BMD (Table 4), the impacts of NOx on fracture risk reduction may be explained by the metabolic effects on these factors.

NO has been implicated in the inhibition of osteoclast activity possibly through an activation of cyclic guanosine monophosphate (cGMP) production in various tissues [3]. Another biological action of NO is mediated by its superoxide scavenging ability leading to the protection of excess tissue oxidation. NO is elaborated by NO synthase (NOS) located in vessels, neurons, and bone marrow cells. However, NO is very labile and quickly converted to NO_2_^−^ or NO_3_^−^ as a receiver of oxygen radicals. Therefore, the role of NOx in fracture risk reduction should be explored in terms of these two metabolic pathways, one is a player as an anti-resorber and the other is a scavenger of superoxide. We have reported that the degeneration of collagen cross-links by glycoxidation plays an important role in the bone tissue brittleness [16, 17]. However, in the subjects of this study, we failed to show the significant linkages between serum levels of NOx and homocysteine, which may disturb enzyme-dependent formation of collagen fiber cross-links [18], and urinary pentosidine as well. On the other hand, Foroumandi et al. have reported a significant negative correlation between serum NO level and circulating advanced glycation end products (AGEs) in healthy subjects [20]. The cause of this discordance in the relationship among NOx and AGEs of the literature and ours is unclear, but the differences in subject ethnicity, age and background at healthy state, or the methodology for measuring pentosidine may account for the discrepancy. Our present results indicated that both pentosidine, one of the well-characterized AGEs, and homocysteine were significantly independent fracture risk factors (Table 2: Model 3 and 4, respectively), and these results were consistent with our previous studies [16, 17, 21].

In another respect, the relationship between NOx and BMI of our cohort was consistent with the prior literature [22]. As shown in Table 3, quartile analysis revealed significant positive associations of serum NOx with L-BMD (p < 0.001) and BMI (p < 0.001). These relationships were also confirmed by multiple regression analysis presented in Table 4, indicating that NOx is a significantly independent determinant for BMI and L-BMD. In addition, Asl et al. have reported a positive correlation between NOx level and obesity as well [22]. Therefore, the evidence on the association between high circulating NOx and high body weight may be robust. A general consensus had been believed since a few decades ago that obese people tended to have higher BMD values, making fractures less likely. In fact, Fracture Risk Assessment Tool (FRAX) includes BMI for the judgement of 10-year fracture risk based on the meta-analysis of 12 cohorts, which showed a negative correlation between BMI and incident osteoporotic fracture rate [23]. However, our previous reports demonstrated that both over- and under-weight were the risks for fracture occurrence though affecting the different bone sites; that is, underweight was a risk for long-bone fractures, while overweight mainly affected vertebrae [24]. This difference in fracture sites among thin and obese body size may be mediated by adipocytokines, leptin and adiponectin [25]. Our previous reports also provided the evidence that the effect of body weight on fracture did not conform to the linear fashion. Importantly, it is well known that obesity can induce the over-exposure to oxidative stress with low ability for anti-oxidant defense [26].

Kasten et al. reported that NOS inhibition evoked the potentiation of osteoclast activity [27]. Our present results shown in the Table 3 demonstrated that higher serum NOx levels were associated with lower urinary NTx in a marginal level (p = 0.049). Further investigation should be required to confirm the effects of NOx on BTMs in treatment naïve subjects. Bolland et al. reported a negative study regarding the effects of nitrates on bone density and bone turnover in a randomized controlled trial. Neither BMD nor BTMs were affected by the intervention using nitrates [13]. Our present results showed that the patients with the lowest quartile of NOx exhibited higher and more rapid occurrence of incident fractures, and the Cox hazards models demonstrated that lower serum NOx level was an independent significant risk for fractures. Thus, the subjects who need nitrate supplementation to prevent fracture occurrence may exist in a certain population. Future investigations will be required to clarify the patients needing nitrate therapy. Several reasons for lowered circulating NOx have been proposed, namely, diminished eNOS expression [28] or activity [29], increased NO oxidation due to NO quenching by AGEs [30], reduced availability of L-arginine [31], or others in animal models. However, there have been no evidential data in human studies.

This study included several limitations. The study population was limited to relatively small size and only Japanese women. Therefore, the present results have to extend into general population including both sexes and various ethnicities. Since the present investigation was carried out in a subset of Nagano cohort study population, possible selection bias might exist. However, baseline data of the current study were almost the same as those of our previous reports in terms of participant age, body composition, and BMD [14–18, 21, 24, 25]. Furthermore, these basic data were comparable with those in Japanese general population, as judged from propensity score [32]; thus, the possible selection bias was unlikely. Some of the patients were observed as under treatment of osteoporosis owing to the ethical reasons, indicating that the underestimation of NOx effect on fracture prevention may exist.

In summary, we clearly demonstrated that the lower serum NOx was an independent osteoporotic fracture risk through lower BMI and BMD, possibly accompanied with high bone turnover. Circulating nitrites and/or nitrates may play a significant role in the occurrence of osteoporotic fractures.

## Supporting information

S1 File This file contains all the supporting tables. (DOCX)
