# Supplementary material for: Nitric oxide is associated with fracture risk in Japanese women
Source: PLoS One. 2023 Feb 7;18(2):e0280854. doi: 10.1371/journal.pone.0280854 (PMC9904477; doi:10.1371/journal.pone.0280854)
Supplement: S1 File — (DOCX) [file pone.0280854.s001.docx]

**Supplemental Table 1. Diagnostic efficiency of NOx on incident fracture by the treatment of osteoporosis (AUC by ROC analysis)**

| Population | AUC | P |
| --- | --- | --- |
| Op Treatment No | 0.603 | 0.0012 |
| Op Treatment Yes | 0.585 | 0.0421 |
| Total Population | 0.595 | 0.0001 |

**Supplemental Table 2. Baseline characteristics in the subjects with higher or lower serum NOx levels**

| Item | Lower group (≤ 74.2 μmole/L) | Higher group (74.3 μmole/L ≤) | P |
| --- | --- | --- | --- |
| Age, years | 68.2 ± 11.3 | 67.1 ± 10.6 | 0.084 |
| Body weight, kg | 49.3 ± 8.2 | 52.1 ± 8.9 | < 0.001 |
| Body height, cm | 150.1 ± 7.1 | 151.5 ± 6.1 | 0.004 |
| BMI, kg/m^2^ | 21.9 ± 3.3 | 22.7 ± 3.3 | < 0.001 |
| L-BMD, g/cm^2^ | 0.906 ± 0.195 | 0.950 ± 0.195 | 0.002 |
| Albumin, g/dL | 4.2 ± 0.3 | 4.3 ± 0.4 | 0.005 |
| PTH, pg/mL | 39.4 ± 19.0 | 41.8 ± 17.7 | 0.094 |
| 25(OH)-vitamin D, ng/mL | 20.5 ± 5.9 | 20.0 ± 5.9 | 0.307 |
| NTx, nmole/mgCr | 52.5 ± 30.0 | 50.0 ± 30.9 | 0.305 |
| Pentosidine, pmole/mgCr | 49.2 ± 39.7 | 45.0 ± 26.3 | 0.100 |
| Homocysteine, nmole/mL | 9.4 ± 39.7 | 9.2 ± 3.6 | 0.579 |
| Prevalent fracture, yes | 38.6% | 29.0% | 0.004* |
| Vertebral osteoarthritis, yes | 59.2% | 56.6% | 0.118* |
| Incident fracture, yes | 41.5% | 25.6% | < 0.001* |

*: χ^2^ test

**Supplemental Table 3. Relationship between vertebral osteoarthritic changes and age, BMI, L-BMD, or NOx at the baseline**

| Item | G0-1 | G2 | G3 | G4 | P |
| --- | --- | --- | --- | --- | --- |
| n | 371 | 337 | 121 | 42 |  |
| Age, years | 62.5 ± 10.0 | 69.3 ± 10.3 | 75.6 ± 7.9 | 73.4 ± 7.5 | < 0.001 |
| BMI, kg/m^2^ | 21.7 ± 3.3 | 22.9 ± 3.4 | 23.2 ± 3.2 | 22.2 ± 2.6 | < 0.001 |
| L-BMD, g/cm^2^ | 0.915 ± 0.189 | 0.923 ± 0.198 | 0.986 ± 0.189 | 1.080 ± 0.189 | < 0.001 |
| NOx, μmole/L | 110.3 ± 66.4 | 116.1 ± 82.4 | 133.0 ± 129 | 110.7 ± 95.7 | 0.087 |
| Prevalent fracture rate, % (n) | 21.3 (79) | 38.3 (129) | 48.8 (59) | 28.6 (12) | < 0.001* |
| Incident fracture rate, % (n) | 20.0 (74) | 37.4 (126) | 40.5 (49) | 42.9 (18) | < 0.001* |

Osteoarthritic change in vertebrae was estimated by the Kellgren-Lawrence grading.

*: χ^2^ test
